# Antiovulatory and abortifacient effects of *Areca catechu* (betel nut) in female rats

**DOI:** 10.4103/0253-7613.70350

**Published:** 2010-10

**Authors:** Jyoti Shrestha, Tara Shanbhag, Smita Shenoy, Arul Amuthan, Krishnananda Prabhu, Stuti Sharma, Samik Banerjee, Sajala Kafle

**Affiliations:** Department of Pharmacology, Kasturba Medical College, Manipal, Manipal University, Karnataka, India; 1 Department of Biochemistry, Kasturba Medical College, Manipal, Manipal University, Karnataka, India

**Keywords:** Abortifacient, *Areca catechu*, antiovulatory, implantation sites, ovarian cholesterol

## Abstract

**Objectives:**

To study the antiovulatory and abortifacient effects of ethanolic extract of *Areca catechu* in female rats.

**Materials and Methods:**

For antiovulatory effect, ethanolic extract of *A. catechu* at 100 and 300 mg/kg doses was administered orally for 15 days. Vaginal smears were examined daily microscopically for estrus cycle. Rats were sacrificed on 16^th^ day. Ovarian weight, cholesterol estimation, and histopathological studies were done. Abortifacient activity was studied in rats at 100 and 300 mg/kg doses administered orally from 6^th^ to 15^th^ day of pregnancy. Rats were laparotomised on 19^th^ day. The number of implantation sites and live fetuses were observed in both horns of the uterus.

**Results:**

The extract of *A. catechu* showed a significant decrease in the duration of estrus at 100 mg/kg (*P* = 0.015) and 300 mg/kg doses (*P* = 0.002) as compared with control. Metestrus phase was also significantly reduced at 100 mg/kg (*P* = 0.024) and 300 mg/kg doses (*P* = 0.002). There was a significant increase in proestrus (*P* < 0.001) phase. However, diestrus phase was unchanged. Histopathological study of the ovaries showed mainly primordial, primary, and secondary follicles in the test groups as compared to control. There was also a significant (*P* = 0.002) decrease in ovarian weight and a significant (*P* = 0.021) increase in ovarian cholesterol level at 100 mg/kg dose. In the study to evaluate abortifacient effect, the mean percentage of abortion with 100 and 300 mg/kg doses were 75.5% and 72.22%, respectively, which was significantly (*P* = 0.008 and *P* = 0.006, respectively) increased when compared with control.

**Conclusion:**

The ethanolic extract of *A. catechu* at doses of 100 and 300 mg/kg has antiovulatory and abortifacient effects.

## Introduction

Infertility is one of the most common problems with significant medical, psychosocial, and economic implications. There are various factors responsible for male and female infertility. Environmental factors are also responsible like volatile organic solvents or silicones, biological agents (viruses, parasites), physical agents (radiation, exposure to electric shock, excessive vibration, or heat),[1] chemical dust, pesticides;[2] alcohol,[3] smoking,[4] caffeine consumption,[5,6] areca nut chewing,[7] etc. Chewing the mixture of areca nut and betel leaf is a tradition, custom, or ritual, which dates back thousands of years from South Asia to the Pacific region. It has now been established that areca nut use causes variety of health hazards including precancerous lesions in mouth. Use of areca nut is common among female population also. Areca nut is most commonly chewed as a constituent of betel quid. The betel quid is a mixture of betel leaf, areca nut, and slaked lime (calcium hydroxide). The important constituents of areca nut are tannins (15%), gallic acids, oily matter (fat 14%), gum and three main alkaloids arecoline (0.07%), arecaine (1%), and guracine, which have the property of vasoconstriction. Arecaidine, guvacoline, guvacine, and choline are present in traces only. Pharmacological actions of arecoline resembles that of muscarine and pilocarpine. Dried areca nut has hepatoprotective,[8] hypoglycemic,[9] astringent, vermifugal, sialogogue,[10] antibacterial, antioxidant, antiseptic, bronchostimulant, euphorient, and wound-healing properties.[7] In addition, it has abortifacient, anti-implantation, and antifertility activities.[11] Studies have revealed that areca nut in male albino rats of Wistar strain causes morphofunctional changes such as stimulation of hormonogenesis and disruption of spermatogenesis.[12] A survey of literature revealed that antifertility effect of areca nut has not been documented in female albino rats. Hence, this study was undertaken to evaluate antiovulatory and abortifacient effects of *A. catechu* in female albino rats.

## Materials and Methods

### Plant material and extraction[13]

The nuts of *Areca catechu* were purchased from local market, chopped into small pieces, and dried under the sun for few days. The dried chopped nuts were powdered and defatted in petroleum ether. The residue was hot extracted in soxhlet apparatus (Tensil Glass Works, Bangalore, India) using 400 mL of 70% ethanol (Qualigens Fine Chemicals, Mumbai, India) for five cycles. This extract was filtered, lyophilized, and concentrated over a water bath to obtain dried extract. This extract was stored in a desiccator (Quality Traders, Eranakulum, Kerala). A suspension of the extract in 4% gum acacia (Nice Chemicals, Cochin, India) was used for the study.

### Acute toxicity study

Acute toxicity was done in female albino rats of Wistar strain weighing 150–200 g. Rats were fasted overnight. They were divided into six groups of two rats each. The alcoholic extract of betel nut was administered through the feeding tube to each group in ascending and widely spaced doses, namely 10, 30, 100, 300, 1000, and 3000 mg/kg (p.o.). The rats were observed continuously for 2 h and then occasionally for further 4 h, and finally overnight mortality was recorded.[14] No signs of toxicity were observed even with 3000 mg/kg of ethanolic extract of *A. catechu*. Therefore, two doses of the extract were chosen, 300 mg/kg corresponding to one-tenth of the maximum tolerated dose (3000 mg/kg) and the other was 100 mg/kg.

### Animals

The experimental protocol was approved by Institutional Animal Ethics Committee. The rats were maintained under standard conditions in animal house approved by the Committee for the purpose of Control and Supervision of Experiments on Animals (CPCSEA). The temperature was 23 ± 2°C and humidity was 50 ± 5%. Mature, healthy female albino rats weighing 150–200 g were used. They were provided with standard rat feed (Amrut lab animal feed, Pranav Agro Industries Ltd., Sangli, Maharashtra) and water *ad libitum*.

### Estrus cycle

**Figure 1:**
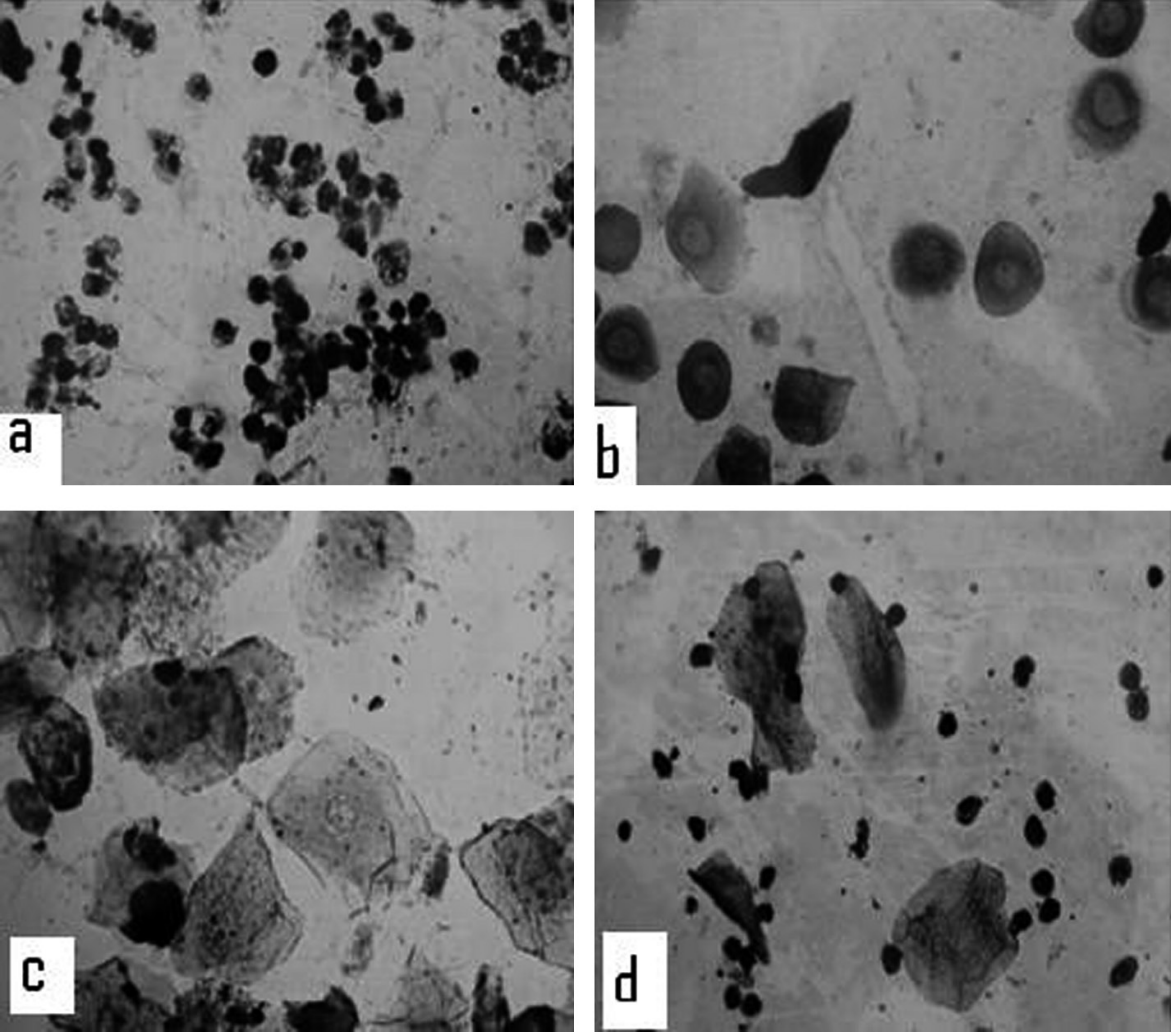
Vaginal smear of rat showing different phases of estrus cycle: (a) Diestrus, (b) proestrus, (c) estrus, and (d) metestrus.

Phases of estrus cycle of each female rat used for study of antiovulatory and abortifacient effects were determined by observing vaginal smear daily between 9 and 10 AM continuously for 15 days. Vaginal smear was prepared by introducing a drop of distilled water into the vagina with the help of a dropper, collecting back and placing it on a clean slide after adding a drop of glycerin.[15] The prepared smear was examined microscopically under low power for different types of cells [Figure 1]. There are four phases in estrus cycle of rat. If majority of cells are leucocytes, then it was labeled as in diestrus phase [Figure 1a]. Presence of large number of nucleated cells indicated proestrus phase [Figure 1b]. Estrus phase was confirmed when the smear showed more than 50% cornified epithelial cells [Figure 1c]. Metestrus phase was indicated by the presence of many neutrophils and scattered squamous epithelial cells in the smear[14] [Figure 1d]. Rats exhibiting three consecutive regular estrus cycles were chosen for the study.

### Antiovulatory effect

This was studied by observing the effect of *A. catechu* on the duration of phases of estrus cycle, estimation of ovarian weight and cholesterol. The rats with three regular estrus cycles were divided into three groups of six each. Rats were grouped in such a way that all the groups were in estrus phase at the start of treatment. Rats in Group I received 2 mL of 4% gum acacia (p.o. daily) for 15 days and served as control. Groups II and III received ethanolic extract of *A. catechu* (p.o. orally) at 100 and 300 mg/kg body weight, respectively, for 15 days. Daily vaginal smears were observed in the morning between 9 and 10 AM. The number of days spent in each phase by each rat was calculated. Then average number of days spent in each phase by each group was calculated. At the end of the study, i.e. on the 16^th^ day, 24 h after the last dose, all the rats were killed by cervical dislocation.[16] Ovaries were dissected out, freed from extra deposition, and weighed. Right ovary from each rat was processed for cholesterol estimation and another for histopathological study.[17]

### Cholesterol estimation

Ovarian tissue was weighed and homogenized in cold saline to get uniform suspension (1:20 ratio that is 20 µL of saline was added per milligram of tissue). Cholesterol was estimated using manual kit supplied by Aspen Laboratory Pvt. Ltd., Delhi. Values were expressed as microgram/milligram of ovary.

### Abortifacient effect

Rats exhibiting three consecutive regular estrus cycles were chosen for the study. The female rats in proestrus phase were mated with male rats of known fertility in the ratio of 2:1 in the evening. Vaginal smears were examined in the following morning for the presence of sperms to confirm mating. The pregnancy rate was 100%. Female rats exhibiting thick clumps of spermatozoa in the vaginal smear were chosen for the study and that day was considered as day one of pregnancy. Eighteen pregnant rats were divided into three groups of six each. Group I served as a control, which received 2mL of 4% gum acacia (p.o. daily). Groups II and III received ethanolic extract of *A. catechu* (p.o. daily) at 100 and 300 mg/kg body weight, respectively, from 6^th^ to 15^th^ day of pregnancy which is the period of organogenesis. On 19^th^ day of pregnancy, all the rats were laparotomised under light ether anesthesia. The number of implantation sites and live fetuses were noted in both horns of the uterus.[16] The observations of the drug-treated groups were compared with control group.

Percentage of abortion was calculated by the formula:

$$\mathrm{Percentage}\;\mathrm{of}\;\mathrm{abortion}\;=\;\frac{\left(\mathrm{Number}\;\mathrm{of}\;\mathrm{implantations}\;-\;\mathrm{Number}\;\mathrm{of}\;\mathrm{live}\;\mathrm{fetuses}\right)\;\times \;100}{\mathrm{Number}\;\mathrm{of}\;\mathrm{implantations}}$$

### Statistical analysis

Results were expressed as mean with standard deviation for data following normal distribution and median with interquartile range for skewed data. *P* < 0.05 was considered statistically significant. The differences between experimental groups were compared using one-way ANOVA followed by Tukey’s *post hoc* test and also Kruskal–Wallis *H*-test followed by Mann–Whitney test with Bonferroni correction for skewed data.

## Results

### Acute toxicity studies

Mortality and behavioral changes were not observed in the acute toxicity study till the dose of 3000 mg/kg. Therefore, two doses (100 and 300 mg/kg) were selected for the study.

### Antiovulatory effect

#### Effect on the estrus cycle

The number of days the rats spent in *proestrus* phase in groups II and III was significantly (*P* < 0.001) higher as compared with the control group. There was also a significant decrease in the median duration of the *estrus* at 100 mg/kg (*P* = 0.015) and 300 mg/kg doses (*P* = 0.002) as compared to the control. *Metestrus* phase was also significantly reduced at 100 (*P* = 0.024) and 300 mg/kg doses (*P* = 0.002) as compared to the control. However, there was no significant change in the duration of the *diestrus* phase between the groups as shown in Table 1.

**Table 1:** Effect of ethanolic extract of *A. catechu* on the duration of the different phases of estrus cycle in rats

*Group*	*Treatment*	*Dose*	*Number of days spent in each phase*			
**	**	**	*Proestrus*	*Estrus*	*Metestrus*	*Diestrus*
I	Control	2 mL	2.00 ± 0.26	3.50	4.50	5.00 ± 0.26
	(4% gum acacia)			(3.00, 4.00)	(3.75, 5.25)	
II	Ethanolic extract	100 mg/kg	5.50 ± 0.50*	2.0 **	2.0 ****	4.83 ± 0.31
				(1.00, 3.00)	(2.00, 4.00)	
III	Ethanolic extract	300 mg/kg	6.50 ± 0.43*	1.0 ***	2.0 ***	5.17 ± 0.31
				(1.00, 2.00)	(1.75, 2.25)	
*P* value	One way ANOVA		<0.001			0.727
	Kruskal–Wallis H test			0.003	0.005	

Values are mean ± SEM in proestrus and diestrus; Values are median (25^th^, 75^th^ percentile) in estrus and metestrus; In estrus and metestrus, Bonferroni correction was done, *P* < 0.025 is considered as significant; n = 6 in each group;

* *P* < 0.001 when compared with control;

** *P* = 0.015 when compared with control;

**** *P* = 0.024 when compared with control.

*** *P* = 0.002 when compared with control;

#### Effect on ovarian weight (right ovary)

In drug-treated groups (100 and 300 mg/kg doses), there was a significant decrease (*P* = 0.002) in ovarian weight as compared with the control as indicated in Table 2.

**Table 2:** Effect of ethanolic extract of *A. catechu* on ovarian weight and cholesterol level in rats

*Group*	*Treatment*	*Dose*	*Weight of right Ovary (mg)*	*Cholesterol level in right Ovary (mcg/mg of ovary)*
I	Control			
	(4% gum acacia)	2 mL	38.00 (36.00, 49.75)	2.98 ± 0.68
II	Ethanolic extract	100 mg/kg 26.50	26.50 (25.25, 27.25)*	5.55 ± 0.59**
III	Ethanolic extract	300 mg/kg	16.00 (13.25, 19.50)*	4.23 ± 0.53
*P* value	One way ANOVA			0.028
	Kruskal–Wallis H test		<0.001	

Values are median (25^th^, 75^th^ percentile) in weight of ovary; In ovarian weight, Bonferroni correction was done, *P* < 0.025 is considered as significant; Values are mean ± SEM in cholesterol; n = 6 in each group;

* *P* = 0.002. vs. control;

** *P* = 0.021. vs. control.

#### Effect on ovarian cholesterol level (right ovary)

The mean ovarian cholesterol level in group II (100 mg/kg dose) showed a significant increase (*P* = 0.021) when compared with the control group, but there was no significant change in cholesterol level in group III (300 mg/kg dose) as shown in Table 2.

#### Effect on histopathological changes (left ovary)

**Figure 2:**
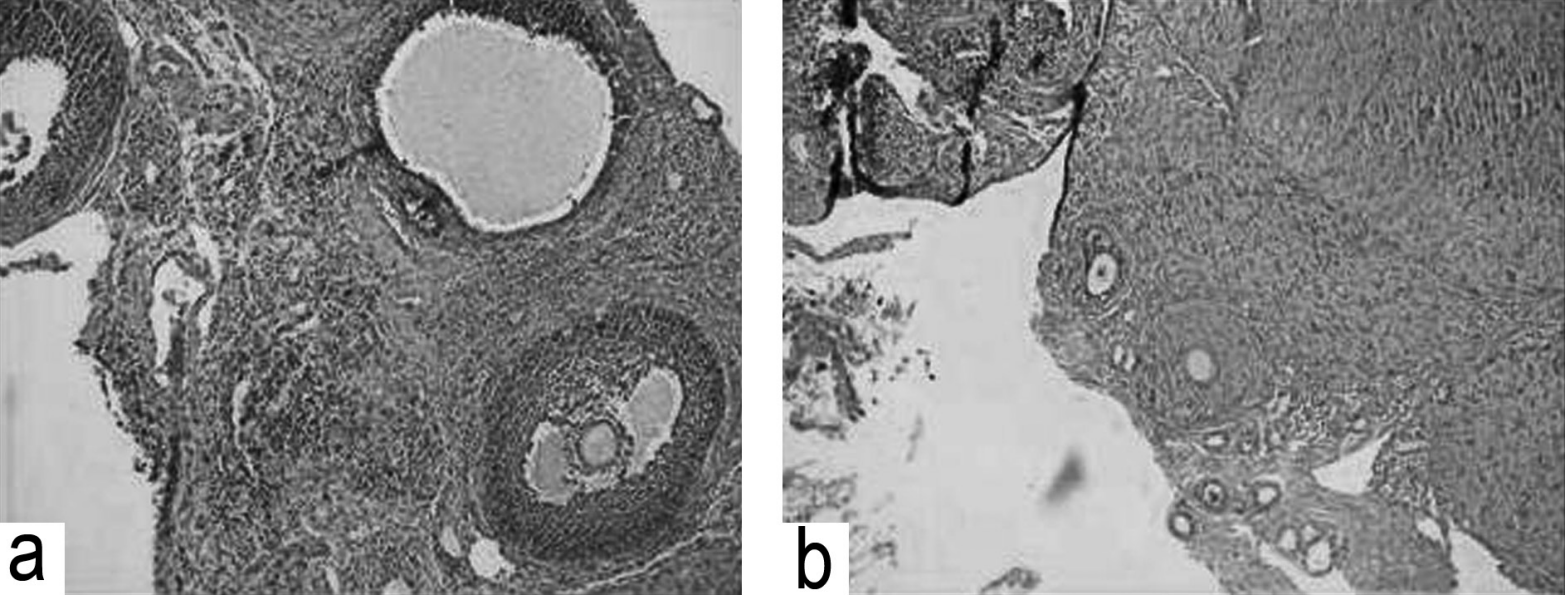
(a) Section of rat ovary in control group showing mature graafian follicle and developing follicles. (b) Section of rat ovary in group II showing primordial and primary developing follicles.

Section of the ovaries in the control group showed primordial, primary, graafian, ruptured follicles, and corpus luteum in the ovarian stroma along with blood vessels, adjacent fibrous areas with mature adipose tissue [Figure 2a]. In group II, ovarian sections showed only primordial, primary and secondary follicles but were devoid of graafian and ruptured follicles [Figure 2b]. In group III, 50% of the ovarian sections had histopathological features same as in control group and remaining 50% were devoid of graafian follicles, ruptured follicles, and corpus luteum.

### Abortifacient effect

**Figure 3:**
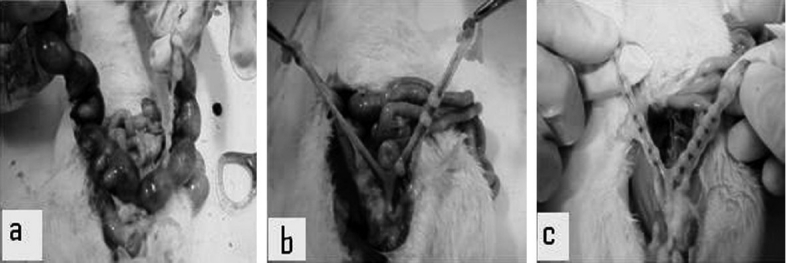
Uterus with the fetuses and implantation sites: (a) control, (b) 100 mg/kg of *A. catechu*, and (c) 300 mg/kg of *A. catechu*.

The mean percentage of abortion with 100 and 300 mg/kg doses were significantly increased (*P* = 0.008 and *P* = 0.006, respectively) when compared with the control group as indicated in Table 3. There was atrophy and a decrease in the number of fetuses in the drug- treated groups [Figures 3b and 3c].

**Table 3:** Abortifacient effect of ethanolic extract of *A. catechu* in rats

*Group*	*Treatment*	*Dose*	*Total number of implantations*	*Number of live fetuses*	*Abortion in %*
I	Control				
	(4% gum acacia)	2 mL	12.17 ± 1.33	12.17 ± 1.33	-
II	Ethanolic extract	100 mg/kg	8.33 ± 2.94	2.5 ± 3.33	75.5 ± 33.43*
III	Ethanolic extract	300 mg/kg	8.33 ± 2.34	2.5 ± 2.51	72.22 ± 29.66**

Values are expressed in terms of mean ± SD, *n* = 6 in each group;

* *P* = 0.008 as compared to control;

** *P* = 0.006 as compared to control.

## Discussion

The effect of ethanolic extract of *A. catechu* on the duration of different phases of estrus cycle, ovarian weight, cholesterol level, and histopathology were studied in rats. During estrus cycle many physiological, biochemical, morphological, and histological changes occur in the ovaries. Follicular growth and ovulation are regulated by endocrine (follicle stimulating hormone, luteinizing hormone, and prolactin) and ovarian hormones (such as progestin, estrogen, and androgen). Ovarian hormones are produced by different cell types of the ovary like granulosa cells of the mature follicles and the corpus luteum.[18] Imbalance in these hormones leads to irregularity in the ovarian functions and duration of the estrus cycle.[19]

In our study, the number of days the rats of groups II and III spent in the estrus cycle showed a significant decrease in the median duration of *estrus* and *metestrus* phases (as compared to the control group). Reduction in the *estrus* and *metestrus* phases indicates nonavailability of the matured graafian follicles or nonmaturation of secondary follicles. Therefore, ovulation was inhibited. There was an increase in the mean number of days the rats spent in *proestrus* phase in groups II and III, which was significant as compared with the control group. The prolongation of the *proestrus* phase indicates that maturation of the follicles in the preovulatory phase was delayed leading to nonmaturation of graafian follicles. This was due to nonavailability of estrogen produced by the granulosa cells which is essential for the maturation and differentiation of the ovarian follicles, or imbalance in the endogenous steroid, protein, and hormones. This result was further supported by the histopathological studies [Figure 2b], in which the transverse section of the ovaries in the drug-treated groups showed the presence of mainly primordial follicles, primary follicles, and secondary follicles in the ovarian stroma along with dilated blood vessels, fibrous, and adipose tissues. In the control group, ovarian sections showed in addition to above, graafian follicles, ruptured follicles, and corpus luteum, which indicates that ovulation has taken place and there was no hormonal imbalance [Figure 2a].

Ovary consists of aggregation of three endocrine tissues, the stroma, the follicle, and corpus luteum. The weight of these tissues constitutes the net weight of the ovary. During the estrus cycle, the weight of the ovarian tissue increases under the influence of gonadotrophic and steroidal hormones. There was a significant decrease in the weight of ovaries in groups II and III as compared with the control group, which indicates a decrease in the activity of the stroma, the follicle, and the corpus luteum in the ovary. This decrease may be due to the nonavailability of gonadotrophic or steroidal hormones or both.[20]

Cholesterol is the precursor for the steroidogenesis of ovarian endocrine tissues, estrogens, progestins, and androgens. The significant increase in ovarian cholesterol in group II when compared with the control group, indicates that cholesterol is not utilized for steroidogenesis.[20] There was no significant increase in the ovarian cholesterol level in group III as compared to control group.

Insufficient progesterone secretion by the corpus luteum or placenta, also termed a luteal phase defect, has been suggested to cause abortion.[21] Therefore, this could be a possible cause for the significant increase in percentage of abortion and abortifacient effect seen in drug-treated groups. Habit of chewing betel nut may cause abortion or disturb normal gestation. Hence, awareness needs to be developed among female populations about the health hazards of betel nut.

## Conclusion

All the parameters in this study showed that the alcoholic extract of *A. catechu* has antiovulatory and abortifacient effects.
